# Practical Observations on Diseases of the Anus and Rectum

**Published:** 1831-01-01

**Authors:** 


### CLINICAL REVIEW,

OR,

### HOSPITAL REPORTER;

WITH

CRITICAL COMMENTARIES.

XLII.

### DUBLIN HOSPITALS.

Practical Observations on Certain Diseases of the Anus and Rectum. By A. Colles, M.D., &c.*

There is a contribution from Dr Colles on the diseases of the rectum, in the last volume of the Dublin Hospital Reports, which is not undeserving of attention. As Dr. Colles observes, this subject is one of high practical interest, and one on which we are not so well informed as could be wished. In fact a *very* good description of the diseases of the rectum and anus is still a desideratum. The topics adverted to by our able author are organic stricture—spasmodic stricture—vascular tumour—and ulcer of the rectum.

I. Organic Stricture of the Rectum. It spares neither age nor sex; it is most frequent about the meridian of life, but Dr. Colles has seen it as early as the seventh or eighth year. He has not witnessed it at or beyond the sixtieth.

" In some few cases the patient appears to be aware of the moment of the first attack of this disease; for he tells us, that without any previous illness, the bowels at a certain period suddenly became costive; that for the purpose of relieving them he took large and repeated doses of physic for three, four, or five successive days; that at length his bowels suddenly gave way and a very severe purging took place, which having continued for a day or two, was

* Dublin Hospital Reporter, Vol. V.

then succeeded by those symptoms which attend the disease when fully formed.

Many patients, however, cannot give any account of the first approach of this disease; they merely state that they have been for many weeks, months, or years, subject to it; that the symptoms from the commencement were pretty much the same as those they now labour under, but perhaps not quite so severe and urgent.

When organic stricture is fully formed, (by questioning him,) we learn from the patient, that in the course of each day he has many and sudden calls to stool; that at each of these he is obliged to strain very much, and that the straining, which is not followed by any severe pain, produces a discharge of not more than a table-spoonful of mucus, which is sometimes streaked with blood, and very rarely mixed with a small quantity of fæces; that these evacuations are generally attended with a copious discharge of wind, and that as soon as the evacuation has taken place, he feels free from pain or uneasiness: the remission however is of short duration, as he is soon again compelled to undergo the same unavailing distress. The number of these discharges are seldom less than from seven to twelve during each day and night; they do not take place at regular intervals, but generally a considerable number occur in quick succession, and then are followed by a pretty long interval of ease. The greater number of these evacuations are devoid of fæces; a feculent stool is passed perhaps once in two or three days; the fæces are then found passed in short pieces and of very reduced dimensions, not larger than a full-sized catheter, and in quantity not equal to what is passed in an ordinary evacuation by a person in health, yet after each feculent stool the patient feels much relieved.

There is not the slightest prolapsus ani with any of these evacuations.

The bladder is in some cases slightly affected; the patient then complains of a little difficulty or delay in passing his urine.

In some cases a fistulous opening forms in the nates or the perinæum, which will admit a probe to pass into the rectum; it yields a moderate quantity of healthy pus. The fistula undergoes little or no change from the time of its first appearance, even until the death of the patient. In some cases, especially in females, I have known the number of these fistulous openings to amount to twelve or twenty. The majority of cases of stricture of the rectum, however, are unattended by fistulæ."

Although this state of daily suffering may proceed for years, yet the functions and appearances of the patient shew little deviation from health. After a lapse of time, which varies in different cases, a peculiar pallor of countenance and wasting are observed, and these are quickly followed by night sweats, and uneasiness in the sigmoid flexure of the colon, which soon extends along the left colon. In females a communication is frequently formed between the rectum and vagina, the greater part of the fæces passing through the latter. In men, but more rarely, a similar communication is established between the rectum and bladder.

As the disease draws towards its close the distress from the state of the rectum is more serious, and on the slightest exertion, coughing, sneezing, or making water, there is an involuntary discharge of a thin brownish fluid of a muco-purulent character. After some time this discharge comes away in the morning the moment the patient rises from his bed. In the last stage of the disease the discharge is incessant and passed unconsciously, and the fæces can only be voided in a perfectly liquid state. The general health declines proportionally; the appetite fails, there is urgent thirst, emaciation is rapid, hectic fever is fully formed, the night sweats are profuse, and the patient appears to be carried off as much by the exhausted state of his constitution, as by the torments of the local disease. Sometimes, in the advanced stage of the disease the patient is suddenly cut off by symptoms of peritoneal inflammation. On examination we find that ulceration had opened the intestine immediately above the stricture, and that through the opening a portion of fæces had escaped into the cavity of the abdomen. In a few patients warm weather seemed to arrest the hectic fever, but it returned and ran its course with greater severity on the approach of Winter.

"Among a considerable number of patients afflicted with this disease, I have had an opportunity in two instances only, of meeting with it in its incipient state. In both of these, the patient complained of different symptoms of irritation of the rectum, frequent stools, discharges mixed with mucus, and certain feelings of uneasiness: on examination by the finger, a thickening and slight projection of the gut was felt at a small spot on one side; this morbid alteration spread gradually round the entire of the canal, and extended along it only to a small distance; but until the morbid derangement of structure had almost entirely performed the circle of the intestine, the patient did not exhibit those symptoms which I consider as the common and inseparable attendants on stricture of the rectum. However constant in their attendance, or unvarying in their course, may be the symptoms of this disease, yet will the surgeon desire to be confirmed in his opinion by manual examination. Proceeding to make this examination, we often observe at the orifice of the anus the following appearance, which is indeed almost always present when the disease is seated near to the external sphincter, namely, at each side of the anus a small projection, which on its external surface appears as a mere elongation and thickening of the skin, but internally presents a moist surface, not exactly like the lining membrane of the gut, nor yet can we say that it is ulcerated; these two projections lie close together below and divaricate above, presenting a resemblance to the mouth of an ewer. Whenever this external appearance exists, I feel almost certain of finding a stricture of the rectum before the finger is pushed as far as the second joint into the gut. In some cases, however, this external mark has not been present."

When the stricture is situated pretty high up, the gut, between it and the anus, is perfectly healthy, but the narrowness of the stricture will barely admit the point of the finger. If it be pushed on with a slight degree of force and a boring motion, it is passed through the thickened and indurated part, and the gut above is felt to be healthy. The extent of the stricture is very variable; sometimes it is little more than a mere ring, at others it stretches along the canal as high as the finger can reach. Dr. Colles has not met with any instance in which the intestine was strictured only by bands thrown across, and he presumes that such must be very rare. In a few instances the stricture was so high as to be barely touched with the point of the finger; on desiring the patient to "force down" a satisfactory examination could be made.

The morbid anatomy of this disease presents all the coats of the intestine, except the peritoneal, very much thickened; the mucous coat moreover is hardened and raised into irregular ridges or folds, but without any ulceration.

The symptoms are so unvarying and the examination by the touch so satisfactory, that the disease can scarcely be confounded with any other. But still there are affections with which it has some characters in common, and from which it would be well to distinguish it with clearness.

1. *Cancer of the Rectum.*—This has the same narrowing of the canal and hardness of its walls, the frequent straining stools and discharge of bloody purulent mucus. But the patient's aspect is leaden and sallow, and he suffers severely from lancinating pains darting through the hips and pelvis into the groins, and down the thighs and legs. If we repeat the examination by the finger after an interval of a few weeks we find that the cancerous ulceration has destroyed some portion of the hardened wall of the intestine, and produced a condition of the parts very different from that of stricture of the same duration.

2. There is a rare form of scirrhus of the uterus and vagina, in which the latter passage is almost obliterated by a cancerous thickening of its walls. Examination by the finger dispels all doubts of the nature of this affection.

3. Enlarged prostate gland may simulate the symptoms of stricture. Examination is a sufficient test.

4. Stricture of the rectum in its early stage may be mistaken for ulcer of the intestine. If an ulcer be low down it will be seen by expanding the anus, or by introducing a blunt polished gorget into the bowel, with its concavity towards the diseased side of the rectum. The finger too, if steadily pressed against an ulcer, will be received into its cavity, although in a hasty examination it feels like a ridge. In ulcer of the rectum there is a severe pain attends the evacuation of the bowels, in stricture there is none.

5. A tumour in the pelvis may compress the rectum. But here the projection comes from one side only, and the coats of the intestine at this part retain their healthy structure and their natural softness.

Lastly, we sometimes find that the finger in ano will not discover any canal in the gut, the entire of its calibre above the sphincter being filled up with soft folds of the lining membrane. Such is not a morbid state, and is unattended by any symptoms.

The treatment by bougies is that usually recommended, and apparently on rational grounds. But Dr. Colles has never known them produce a permanent cure, although by making a deep groove run spirally in their whole length, he has rendered them the vehicles for conveying various applications to the seat of the disease. Dr. Colles has tried mercury fully without any lasting benefit; he has also given arsenic, cicuta, iron, with the same result. Large quantities of mucilage appear to afford most relief, and blue pill with twice as much Dover's powder has occasionally given temporary alleviation.

This is the only disease to which Dr. Colles would apply the title of stricture of the rectum. In the course of his practice he has not been able to discover a single case of the spasmodic stricture described by authors, and recommended to be treated by the bougie. He not only doubts the existence of the disease, but can recollect many cases, in which this suspected condition of the rectum has yielded to the ordinary means for improving the state of the stomach and bowels. The assurance to the patient that there is no actual stricture is a measure of paramount importance. Dr. Colles looks on the obstruction and thickening of the colon, either at its junction with the rectum or higher up, as an affection very different in its symptoms and its nature from the stricture of the rectum under consideration. In the advanced stages of the former affection the patient can procure no evacuation without the aid of purgatives, nor is he, as in stricture of the rectum, harrassed by frequent ineffectual strainings at stool; guided by his own sufferings he can point to the spot where the disease is seated. On examination after death we find a very considerable thickening of the coats of the intestine in the circular direction, but of short extent longitudinally. The canal at the seat of stricture is nearly obliterated, while the intestine immediately above is dilated into a wide pouch.

Our author thinks that the symptoms described by authors as indicating spasmodic stricture, or organic stricture seated beyond the reach of the finger are fallacious. We are told, that examination with a soft bougie, the very diminished diameter of the fæces, and the admixture of blood or mucus with the stools, point out the existence of these diseases. The soft bougie, however, will receive an impression from the projecting ridge of the sacrum, for it is no easy matter to prevent it from being arrested at this point. The diminished diameter of the fæces may be produced by any irritation in the rectum, even in perfect health. And, lastly, the admixture of mucus or blood with the fæces is frequently met with in other and very different affections of the bowels.

*Case.* "Mr. ——, æt. ann. 36, who had lived rather fully and freely, applied to me six or seven years ago under the following circumstances: he said that he had been, for the last two years, affected with stricture of the rectum; that in the preceding year he had an attack of dysentery, and that since that time he found the stricture worse; the diameter of the fæces diminished, calls to stool more frequent, and seldom passing without an admixture of blood and purulent mucus; he was losing flesh rapidly, his appetite and rest were very indifferent, and his mind was miserable. Having examined the rectum by the finger, I expressed to him my hopes that he was not affected with such an intractable malady, by which I very nearly forfeited his confidence; for he told me that he had applied to a very eminent physician in the north of England, and then to another in London, both of whom assured him of the existence of this disease; and lastly, he had been under the care of the late Mr. White of Bath, who furnished him with the bougies he was then using, and immediately introducing one, he shewed me the mark which the stricture impressed on it. My most urgent remonstrance could at this time only obtain from him a promise that he would not use the bougie as frequently, or for as long a period as usual. By small doses of blue-pill, combined with compound powder of ipecacuanha and an enema of olei oliv. cum subacet. litharg. liquor., the irritation of the bowels was mitigated in the course of eight or ten days. Availing myself of this favourable change, I again urged him to lay aside the bougie, and with some difficulty obtained a truce for ten days; within this period it fortunately happened that he passed one consistent motion, in which the fæces were of a large diameter: after this I had but little trouble in prevailing on my patient to lay aside altogether the use of the bougie. By persevering in the internal use of mild bitters and bark, and injecting every night an enema, consisting of ol. oliv. and unguent. supernitrat. hydrargyr. into the rectum, his disease was finally cured, and since that time his bowels, though sometimes deranged by ordinary complaints, have never suffered from a return of the former affection, and he has enjoyed very good health."

Dr. C. believes, and it is very probable, that had the patient persevered in the use of bougies, his life would have paid the penalty. The event shewed there was no stricture in the rectum. Our author has known two cases of fatal peritonitis from attempts to dilate a *supposed* stricture at the top of the rectum or termination of the colon, and he has heard, on the best authority, of two others.

Dr. Colles looks on spasmodic stricture of the sphincter ani as a very rare disease. The only case he ever met with had been treated as a stricture of the rectum. The patient, a young man of rank, informed Dr. Colles that during the two last years he had suffered from it for two or three months at a time, and that he enjoyed immunity from it for four or five months. To convince the patient that his fears of stricture were unfounded, our author passed a wooden globe, three inches and a half in circumference, mounted on a stalk of whalebone, ten inches up the rectum, without meeting with any obstruction.

### II. VASCULAR TUMOURS OF THE RECTUM.

These are commonly called "Varicose tumours," "hæmorrhoidal excrescences," but Dr. Colles thinks the terms inappropriate. We need not detail the symptoms they produce, but we may mention an examination which Dr. Colles made of their structure after death. On slitting up the rectum in a patient affected with them, he saw three blood-vessels, each as large as a crow-quill, running for some way down the intestine, and then dividing into a number of branches; these vessels ramified very profusely, and each seemed, by interweaving of its branches, to form one of these tumours. The trunks and branches were covered only by the lining membrane of the intestine. Dr. Colles prefers excision of these tumours, as tetanus, the dreaded consequence of the ligature, cannot be guarded against, whilst hæmorrhage, the only likely unpleasant result of excision, may be prevented and suppressed. The following is his method of operating.

"The tumours having been made to protrude, by means of a purgative injection, I direct my assistant to pass a hook or common tenaculum through one or two of the largest, while I seize another lengthwise with a polypus-forceps, then drawing the tumor a little towards the axis of the gut, with a large pair of scissars passed behind the forceps, I cut off all that portion which is engaged between its blades: I then proceed in the same manner to remove those tumors which the assistant holds transfixed by the hook. By fastening and drawing out the tumor with the forceps, we much facilitate its removal by the scissars. Proceeding in this way I guard against these tumors being drawn up within the sphincter as soon as the first had been removed. I do not think that any case will require the removal of more than three of these tumors, and not unfrequently the cure will be ensured by cutting off only two of them. When the operation is finished, the protruded parts generally retire within the sphincter: should any part remain out, it must be completely pushed in with the finger.

In order to guard against the danger of hæmorrhage, I take care not to prolong my incision higher on the bowel than what I conceive will, when replaced, lie within the circle of the sphincter; for, if we cut the gut higher up, this part, when returned, may bleed freely from not having any surface closely opposed to it. Besides we know that by cutting higher up we are in danger of cutting the trunk of the vessel, instead of confining our incision to the tumour, which is composed solely by the convolutions of its very minute branches."

In a case in which the hæmorrhage was troublesome, Dr. Colles adopted Petit's method of stopping hæmorrhage from the rectum, making use of sponge instead of lint. He would be afraid of cutting away all the protruding tumours, together with the skin at the verge of the anus, on account of the great subsequent contraction of the outlet. In one case in which this was done for warts, the condition of the patient was really miserable. The disease is much alleviated, if not ultimately cured, by an injection of four or five ounces of a solution of the sulphate of zinc in water, gr. ij. ad ℥j. It should be thrown up every night at bed-time, and retained during the night. Dr. Colles has imagined that the plan of treatment succeeded best if resorted to after one of the painful fits of the disease to which patients are subject. He has known many persons benefited by smearing the protruded parts after each evacuation, with a liniment of ol. oliv. ℥ij. liq. plumb. subacet. ʒj.

III. Ulcer of the Rectum.

"We sometimes find within the rectum, at a short distance above the anus, an ulcer unconnected with any other disease. In such cases the patient complains that he observes his linen stained with a purulent discharge, which often flows when he is not at stool; on examination this will prove different from healthy pus, frequently containing an admixture of thin bloody fluid; at times the quantity of discharge is much lessened, and then the sufferings of the patient are aggravated, but on the flowing off of a larger quantity he experiences great relief; he suffers sharp pain on going to stool, and this continues for an hour or two. On examination, the finger soon discovers the seat of the disease, which at first feels raised and rather rough, but by pressing the finger firmly on this spot, the point sinks into a small hollow cup of an ulcer, the edges of which are found in some degree hardened. We may obtain a satisfactory view of the ulcer, by passing upon the finger a blunt polished gorget, the cavity of which is to look towards the seat of the disease; then by everting the anus as much as we can, we shall obtain a full view of the ulcer by the light reflected from the gorget.

The unhealthy nature of the discharge, the degree of pain and hardness of the edges of this ulcer, might make us apprehend some carcinomatous affection; but in any doubtful case, by temporizing and watching the course of the disease for a few weeks, we shall have all doubts removed; for if it be cancerous, the ulcer will in this period, have changed in form and extended in size; besides, on strict examination, whilst the point of the finger lies in the cup of the ulcer, it will be found that the hardness is confined to the edges, whilst the bottom of the ulcer is soft.

The remedy for this disease is to introduce into the rectum a convex-edged scalpel, and make an incision through the entire length of the ulcer, continuing it through the sphincter, and dividing the verge of the anus; as soon as this wound has got into a state of suppuration, we should dress it and the ulcer, with some stimulating ointment introduced on a dossil of lint. The cure goes on without interruption, although it is rather tedious and slow of healing. I need hardly add, that the final cicatrization will be promoted by the occasional application of nitrate of silver."

This concludes the paper of Dr. Colles on a subject which practical men will deem highly interesting. We have given it without any comments of our own, although there are some points on which we quite disagree with our author, and others in which the omissions are considerable. Auy thing which emanates from Dr. Colles is deserving of attention, and we leave our expeiienced readers to form their own opinions and conclusions.

---

